# An Update on Usage of High-Risk Donors in Liver Transplantation

**DOI:** 10.3390/jcm11010215

**Published:** 2021-12-31

**Authors:** Haris Muhammad, Duha Zaffar, Aniqa Tehreem, Peng-Sheng Ting, Cem Simsek, Ilker Turan, Saleh Alqahtani, Behnam Saberi, Ahmet Gurakar

**Affiliations:** 1 Division of Gastroenterology and Hepatology, School of Medicine, Johns Hopkins University, Baltimore, MD 21205, USA; drharismuhammad@gmail.com (H.M.); khanduha16@gmail.com (D.Z.); brian.ting@jhmi.edu (P.-S.T.); cemsimsek90@gmail.com (C.S.); salqaht1@jhmi.edu (S.A.); 2 Department of Internal Medicine, Mercy Hospital, Buffalo, NY 14220, USA; aniqatehreem@gmail.com; 3 Department of Gastroenterology, School of Medicine, Ege University, Izmir 35040, Turkey; ilkerturan@gmail.com; 4 Beth Israel Deaconess Medical Center, Division of Gastroenterology and Hepatology, Harvard Medical School, Boston, MA 02215, USA; bsaberi@bidmc.harvard.edu

**Keywords:** liver transplantation, steatotic donors, split liver, donors after cardiac death, HIV Organ Policy Equity (HOPE) Act

## Abstract

The ideal management for end stage liver disease, acute liver failure, and hepatocellular carcinoma (HCC), within specific criteria, is liver transplantation (LT). Over the years, there has been a steady increase in the candidates listed for LT, without a corresponding increase in the donor pool. Therefore, due to organ shortage, it has been substantially difficult to reduce waitlist mortality among patients awaiting LT. Thus, marginal donors such as elderly donors, steatotic donors, split liver, and donors after cardiac death (DCD), which were once not commonly used, are now considered. Furthermore, it is encouraging to see the passing of Acts, such as the HIV Organ Policy Equity (HOPE) Act, enabling further research and development in utilizing HIV grafts. Subsequently, the newer antivirals have aided in successful post-transplant period, especially for hepatitis C positive grafts. However, currently, there is no standardization, and protocols are center specific in the usage of marginal donors. Therefore, studies with longer follow ups are required to standardize its use.

## 1. Introduction

Ever since Thomas E. Starzl laid its foundation in March 1963, liver transplant (LT) has become the standard of care for end stage liver disease (ESLD), acute liver failure, and selected hepatocellular carcinomas (HCCs) [1]. According to the Scientific Registry of Transplant Recipients (SRTR), 12,767 new registrations were added to the United States transplant list in 2019 [2]. Significantly, there was a spike in the percentage of older (aged ≥ 65 years) candidates by 20.8%, while alcohol-related liver disease increased up to 31.3% and the proportion of non-alcoholic steatohepatitis increased to 35.6%. The severity of liver disease, estimated using the first active Model for End-Stage Liver Disease (MELD), also increased, with an increasing proportion having an initial MELD between 15–24 (36.3%) [2]. Deceased donor liver transplant (DDLT) rate among adult waiting list candidates was 61.3 per 100 waiting list years. Thus, over the years, there has been a continuous rise in LT throughout the US. Concurrently, patients on the transplant waitlist are experiencing increasing deaths due to a lack of donors. This is due to the growing gap between demand and supply. In order to cope with the situation of organ shortage, transplantation centers have expanded their criteria of donor selection, and utilizing marginal grafts is becoming more and more prevalent. Compared to healthy allografts, extended criteria donor livers are more susceptible to ischemia- reperfusion injury and relevant impairment of allograft function upon prolonged cold ischemia time (CIT), and are, therefore, as-sociated with a higher risk of postoperative complications. Thus, simultaneously taking into consideration the waiting time is important [3].

## 2. Donor Grafts Which Are Virally Infected

Due to continuous increase in LT cases, there has been consideration to increase utilization of organs from donor livers which were previously discarded, including virally infected donor livers. This has been largely possible due to effective antivirals that has resulted in better outcomes.

### 2.1. HIV

The first HIV liver transplant conducted by Nor et al. was done in the pre HCV direct acting antiviral (DAA) era. In that study and many other studies of the same era, good short-term outcomes were seen in pure HIV candidates, but patient/graft survival was significantly decreased by the presence of HCV, either as mono infection or combined with HIV [4,5]. Post LT mortality using HIV positive donors was reduced considerably when DAA’s efficacy of almost 100% managed HCV infections both pre and post-transplant [6]. This promising change along with the fact that there has been a global shortage of liver donors, prompted researchers to study HIV positive donor LT vigorously. A study was conducted in 2010 where Dr. Elmi Muller launched HIV positive donor kidney transplants in South Africa [7]. His prospective, nonrandomized study included 27 HIV positive recipients who were followed for a median of 27 months. The 1- and 5-year survival were similar to HIV negative controls at the center (84% vs. 91% and 74% vs. 85%, respectively). Rejection rates were 8% at 1 year and 22% at 3 years [7]. Immunosuppressants used in this study included induction therapy with anti–T-cell antibody and maintenance therapy with tacrolimus, mycophenolate mofetil, and glucocorticoids [7] In another set of studies conducted in Europe, it was shown that with the use of HAART (Highly Active Anti-Retroviral Treatment), HIV could fully be brought under control post-transplant [8]. Results from such studies against a background of soaring organ shortage led to the passing of the HIV Organ Policy Equity (HOPE) Act (passed in 2013 in the USA) which was a breakthrough in the field of transplantation [9]. It allowed HIV positive donors to offer grafts to HIV positive recipients. Calmy et al. reported one of the early successful LT in Switzerland in 2015, where a 53-years-old HIV positive was successfully transplanted from an HIV positive donor [8]. The crucial part was played by antiretrovirals post LT, which helped to maintain undetectable HIV RNA with no graft rejection. A steroid-free immunosuppressive regimen including basiliximab induction, as well as tacrolimus and mycophenolate mofetil was used in this study [8]. From 2016, encouraging results from John Hopkins was reported for HIV positive donor to HIV positive recipient transplantation [10]. A longitudinal study done recently as a part of HOPE pilot trial followed HIV positive recipients who had received HIV positive donor liver and kidney grafts. After 3 years of follow up, no evidence of donor derived HIV superinfection was detected in any of the recipients, including one who had temporarily stopped the HAART therapy [11]. Another survey was done on 209 transplant centers to study center level barriers in implementation, knowledge, attitudes, and planned HIV positive donor protocols [11]. It was deduced that most centers (91.2%) were aware of the legality of HIV positive donor transplantation while 21.4% were oblivious to HIV related guidelines. Furthermore, most centers (83.2%) stood in favor of HIV positive donor liver transplantation. However, they believed the willingness of their HIV positive candidates to accept grafts from HIV positive donor organs could be an issue (*p* < 0.001) [11]. Other factors on top of the HOPE protocol that could determine HIV positive donor transplants were: degree of endemicity of HIV in an area, HIV positive recipient load, and total transplant load [11]. Thus, the HOPE Act made it possible for the implementation of transplantation of HIV positive donors into HIV positive recipients, which would have otherwise been rejected. The HOPE Act also stipulated for the efficient utilization of organs that were earlier claimed to be HIV positive and discarded but actually were only false positives. The Action Trial of HOPE identified certain patients who showed positive HIV serology or NAT (Nucleic Acid Testing), but never had an infection. These were classified as false positives and 10 such patients were identified in the study. From these 10 suspected false positives, 21 HIV positive recipients received transplants. Later, all the donors were found to be uninfected. When juxtaposing these results to the total donors in the US, we can expect such 50 to 100 false positives who could serve as potential donors [12]. The American Society of Transplantation has also issued guidelines for HIV positive recipients who not only suffer from higher wait list mortality but decreased access to transplantation as well. Even though more data is emerging in support of utilization of HIV positive donors in HIV positive recipients, it is still not advisable to use HIV positive allografts in HIV negative recipients as HIV can only be controlled and not cured. In 2017, in Africa, there was an emergency LT for a life-threatening state wherein an HIV negative child received a graft from his mother who was HIV positive. More than a year following LT, no viremia has been reported in the recipient child [13]. Even though the overall results look promising, differences between HIV infections in Africa vs. the US should be kept in mind. Differences in terms of HIV sub type, exposure to HAART, and prevalence of HAART resistance are critical determining factors for HIV positive donor LT outcomes. Thus, more data is needed in support of such transplantation in life saving situations.

### 2.2. HCV

An estimated 300 to 500 additional liver allografts from HCV positive donors could be utilized for transplantation to maximize liver donor pool [14]. Increasing intra venous opioid consumption in society has not only led to a greater incidence of HCV but also overdose related deaths [15]. The US opioid epidemic continues to increase, and deaths from opioid misuse increased threefold between 1999 and 2014 [16]. This unfortunate event has, however led to an increase in successful incorporation of HCV positive donor liver allografts in the donor pool due to availability of effective DAA’s [17,18]. Therefore, shifting the spotlight on making HCV positive donor liver allografts as efficient as possible for LT is the need of the hour so it can help us deal with the organ shortage that we are currently facing. In 2015, the United Network for Organ Sharing (UNOS) made NAT a compulsory test along with serology in donors. Both NAT and serology positive (viremic, sero positives) suggest active infection, which has high infectivity, whereas only NAT positive (viremic, sero negatives) indicates a window period that also has considerable infectivity. A positive serology only (nonviremic, sero positive) could mean either a treated/cleared HCV infection or a false positive. The infection risk for pure sero positive ranges between 0% to 16%, whereas in viremic it is almost 100% [19]. The introduction of DAAs has improved LT outcomes because of increased sustained virologic response and fewer adverse effects. The use of the HCV viremic organ for an HCV negative recipient requires the recipient to have prompt posttransplant access to pan genotypic DAA treatment, and preemptive therapy is recommended [20]. Study by Northup et al. on 934 HCV positive donors suggested that HCV positive liver donors showed no increased mortality risk in HCV positive recipients compared to HCV negative liver donors [21]. Similarly, Ting et al. published results from a cohort of 26 HCV sero negative recipients of HCV seropositive donor grafts followed by preemptive DAA regimen, defined as the initiation of DAA after the first positive HCV NAT in LT recipient (median 5.3 weeks after LT) [18]. All 12 recipients completing their DAA regimen and reaching sufficient follow up achieved sustained virological response. Out of the 12 recipients who achieved sustained virological response, 1 received Ledipasvir/Sofosbuvir for 12 weeks, 1 received Sofosbuvir/Velpatasvir for 8 days, followed by Ledipasvir/Sofosbuvir for 23 weeks, and 10 recipients were treated with Glecaprevir/Pibrentasvir for 12 weeks [18]. While we have an established DAA protocol pre/post-transplant for the former, a proper protocol for the latter is awaited. The timing and duration of DAA treatment are variable among centers. Studies are being conducted, and one involving Kidney Transplants from HCV positive donors into HCV negative recipients have shown promising results wherein 19 such recipients on getting the graft underwent DAA treatment regime for post-transplant viremia and showed complete sustained virological response [22]. In the study by Novak et al. 21 HCV negative recipients were transplanted with HCV (nonviremic, sero positive) positive kidney grafts, none showed NAT positive on follow up and had a survival rate of five years [23]. Also, considering that HCV is now a curable disease, using HCV positive donor grafts in HCV negative recipients seems plausible, especially in the setting of organ shortage that we are facing. A recent publication from Cotter et al., after reviewing Scientific Registry of Transplant Recipients database has shown that only 87 HCV positive to HCV negative transplants were done before January 2018 and they showed similar 2 years survival outcome as for recipients of HCV negative donors [24]. Minimizing other graft limiting factors affecting the transplant outcomes, from HCV positive donor, may further improve the success rate. One such factor being allograft fibrosis for which an algorithm was suggested in a study. It showed that the stage of hepatic fibrosis in NAT positive donors further determined the suitability of using the graft. On biopsy (done after NAT was positive), liver fibrosis greater than Stage 2 rendered the graft to be declined considering significant allograft fibrosis [25]. A recent meeting consensus report has recommended that young HCV viremic donors (<35 y of age) are likely to have minimal fibrosis and do not require pre donation liver biopsy, and can have a surgical assessment at the time of organ procurement to determine if a liver biopsy is indicated. Older HCV viremic donors (≥35 y of age) with chronic infection should undergo a liver biopsy to exclude those with advanced (F3 or F4) fibrosis. Mild fibrosis (F2 or lower) is acceptable for transplantation [20].

Another modifiable marginal factor is the donor age. With an, unfortunately, higher incidence of opioid overdose deaths, liver grafts are now coming from younger donors, since opioid use is more common in this age group [26]. Hence, close post-transplant follows up coupled with the immediate commencement of DAA as soon as HCV is detected is the best strategy that can not only ensure successful utilization of HCV grafts but also deal with a rare, yet severe complication of HCV, namely fibrosing cholestatic hepatitis which can cause rapid graft failure. DAA regimen has significantly improved the management of HCV even in post-transplant and immunocompromised states. This has made use of HCV positive grafts much more acceptable and even greater than that of HBV and HIV positive donor grafts. Unlike HCV, latter diseases can only be suppressed and not cured. Despite DAA’s efficacy in the face of HCV positive donor transplant, some patients who do not have a very high MELD score are not treated with DAA. This is because getting cured from HCV post DAA therapy causes a reduction in their MELD score which takes away their eligibility status for a transplant. Such a category is often called MELD purgatory. HCV positive donor grafts can specifically be used in the case of MELD purgatory patients with decompensation who even though are deemed ‘too fit’ for a transplant due to a low MELD score, can get an HCV positive donor graft to improve their quality of life. Even though more evidence is in favor of HCV positive donor grafts, some of the challenges we are facing are unwillingness of patients to receive an HCV positive graft, ethical problems especially when viewing the scenario as a conscious transference of infection in a patient and cost factors/insurance coverage for DAA. Despite the challenges, using HCV positive donor grafts is a plausible option now being used in most transplant centers.

### 2.3. HBV

Around 0.24 billion people in the world are positive for hepatitis B surface antigen (HBsAg) [27]. In the current era, using hepatitis B immune globulin (HBIG) and antivirals post transplantation has decreased the incidences for recurrence of HBV and improved overall survival [28]. Nonetheless, the transmission of infection is a major concern, and it has not only been reported for hepatitis B surface antigen (HBsAg) positive donors but also for liver donors who were negative for HbsAg but anti-hepatitis B core antigen (anti Hbc) positive. HBcAb positive donors can have covalently closed circular DNA (cccDNA) retained inside hepatocytes leading to risk for HBV transmission. Historically, the first HBV positive donor graft was done in pre-antiviral era on a child in need of an emergent liver transplant for a life-threatening condition. Several months later, the child tested positive for HBV which was taken care by lowering his dose of immunosuppressant drugs and using ciprofloxacin along until he tested negative [29]. A systematic review including 39 studies reported considerably lower risk of de novo HBV infection in anti HBc positive (15%) compared to HBV naive recipients without prophylaxis (48%) who received liver grafts from anti HBc positive donors [30]. Similarly, Skagen et al. reported de novo HBV rates in recipients of anti HBc positive grafts as follows: 18% in previously vaccinated recipients, 14% in isolated anti HBc positive recipients, and 4% in anti HBc and anti HBs positive recipients in the absence of post LT prophylaxis [31]. However, the risk of de novo HBV infection for both HBV naive (from 48% to 12%) and HbcAb/HbsAb positive recipients of HbcAb positive grafts is reduced (from 15% to 3%) by post LT prophylaxis with HBIG and lamivudine [30]. Currently, HBIG prophylaxis is not recommended in HBsAg negative recipients regardless of the presence or absence of anti HBc and/or anti HBs [32]. When prophylaxis is used, HBV viral burden at the time of transplantation should be the determination factor of dosing and duration of HBIG [16].A meta-analysis study showed that the pooled risk of HBV transmission in HbsAb positive patients (vaccinated or resolved) receiving HbcAb positive donor grafts was similar whether or not they were on prophylactic antiviral treatment post transplantation [33]. This well highlighted the protective effect of HbsAb. These studies suggest that prior infection (HbsAb & HbcAb positive) and vaccination (HbsAb positive) have a protective role against HBV recurrence.

Post-transplant HBIG administration has become prevalent in transplant centers along with antivirals, depending on recipient’s risk status to keep HBsAb titers between 100 to 500 IU/mL [34,35]. When compared, vaccination before transplant seems to be an overall better strategy than HBIG. Not just cost effective but they are also convenient and maintain sustained levels of protective HbsAb after transplant, thus decreasing the risk for transmission of HBV. Loggi et al. studied 10 patients (all were hepatitis B core antibody (HBcAb) positive who were transplanted with HBsAg positive donors and followed for 42 months. Results showed that antiviral therapy effectively controlled HBV replication with no signs of hepatitis [36]. A larger study comparing patients who underwent LT with HBsAg positive donors vs. HBsAg negative donors showed comparable results in patient and graft survivals with no difference in complications such as primary non function, acute rejection, and biliary complications [37].

In the use of HBsAg positive donors, the following criteria are recommended: normal liver function profile, donor and recipient HDV negativity, and donor pathology excluding fibrosis or significant inflammation [32]. HBsAg positive grafts should only be used if there is an option for indefinite prophylaxis with entecavir or tenofovir. The benefit of HBIG in HBsAg negative recipients of HBsAg positive grafts is unclear. Anti HBc positive grafts can be used in HDV positive recipients who are treated with antiviral plus HBIG post-transplant [38]. While the potential risk of de novo HCC is not observed in HBsAg negative recipients of anti HBc positive liver grafts who received antiviral prophylaxis post-transplant, this risk is unknown in HBsAg negative recipients who received liver from HBsAg positive donor [32,39].

Some studies have gone as far as setting cut offs for deciding management protocols for preventing donor to recipient HBV transmission. A small-scale study done in 2008 for example, involved four HbsAb positive patients receiving grafts from HbcAb positive donors. These patients received Lamivudine, and on follow up HBV infection was seen in only one patient who happened to have HbsAb titers < 10 IU/L whereas the rest of them who happened to have titers > 10 IU/L showed no HBV infection. So, they suggested that combination treatment was not required if HbsAb titers > 10 IU/L. Another interesting study highlights the role of HBV vaccination in preventing de novo HBV infections in recipients. It suggests that certain levels of HbsAb being actively produced by the body’s immune system post vaccination have a protective role and that vaccines should be given before and after transplant in all patients who are set to receive liver grafts from HbcAb positive donors. The goal of vaccination is to achieve a post operative HbsAb titer of >100 IU/L [40]. This has been shown to be protective against de novo HBV recurrence. This study further went on to say that candidates with pre operative HbsAb levels < 1000 IU/L require Lamivudine as post-transplant prophylaxis and that it can be stopped safely only when post-transplant HbsAb levels > 100 IU/L. Furthermore, patients who have pre transplant HbsAb levels > 1000 do not require prophylaxis. Notably, both cases require booster HBV vaccination after steroid withdrawal [40]. Despite having made considerable progress in this field, certain circumstances remain a contraindication for receiving HBV positive donor grafts. One such condition is the HBV/HDV, co-infected recipients. In Italy, a small-scale study was conducted on 3 HbsAg positive recipients, out of which 2 were co infected with HDV. After receiving HbsAg positive grafts, all 3 showed persistence of HbsAg despite being on HBIG, and the 2 patients who were HDV co infected showed HDV recurrence necessitating re transplantation in 1 of them [38]. Only 1 recipient who was not co infected with HDV showed promising results [38]. The reason seemed to be an HBsAg overload coming from both donor and recipient and which could not be brought under control by HBIG, leading to re activation of HDV and its undesirable outcomes. With time, improvised risk assessment tools have come up for HbsAg positive donor LT. One such tool is the HbsAg titers which are now being regularly used in the clinical scenario. The titers in donor can tell us about the risk of transmission, and in recipient, they tell us about the ‘entity’ of reactivation and help monitor the response towards antiviral drugs. With all the advancements and supporting data, the results look promising. To conclude, incorporating HBcAb positive grafts will further expand pool with reasonable outcomes.

## 3. Donors with Fatty Liver

Steatosis is commonly found in the donors and is classified at the cellular level as macro steatosis or micro steatosis, depending on the pattern of fat infiltration [41]. In macro steatosis hepatocytes, nuclei are pushed to the side, and the cell is covered by one vacuole primarily. It is usually associated with obesity. Whereas, in micro steatosis there are several fat particles and nuclei is in the center. It is likely due to dysfunctional mitochondrial β-oxidation linked to drugs, toxins, or metabolic disorders [42]. Steatosis is also classified based on the percentage of liver cells involved as mild (<30%), moderate (30% to 60%), or severe (>60%) steatosis [43]. Fortunately, even marked micro steatosis is not associated with poor prognosis after LT [44]. Contrary to this, macro steatosis leads to poor graft function [45]. Therefore, transplantation centers usually discourage using livers with >30% macro vesicular steatosis as it is an independent risk factor for graft survival [46]. Graaf et al. did a retrospective study including 184 donor liver biopsies and reported primary non function (*p* = 0.002), early kidney dysfunction (*p* = 0.040), and decreased graft survival at 3 months (relative risk = 12.09 (8.75 to 19.05), *p* = 0.000) and at 1 year (*p* = 0.000) in patients with severe macro steatosis [47]. Early biliary complications were also seen in moderate macro steatosis [47]. In a clinical trial involving 3007 patients, severe donor liver steatosis (>60%) was associated with higher HCC recurrence (adjusted HR 1.65, 1.03 to 2.64; *p* = 0.037) [48]. Recently, a meta-analysis including 16 studies with 3226 donors reported a significant increase in primary nonfunction [(odds ratio (OR): 2.47, 95% confidence interval (95% CI): 1.44 to 4.27), and an increased in trend in 1 month patient mortality (OR: 1.90, 95% CI: 0.98 to 3.71) when using moderately and severely fatty livers, while 1 year mortality was not much influenced [49]. Likewise, Wong et al. retrospectively compared >60% macro vesicular steatotic liver grafts with ≤60% steatotic grafts. They found out that early allograft dysfunction and 30-day mortality, and 1 year and 3-year overall survival rates were similar between the two groups, as long as used with caution [50]. Another study reported that both high BMI and steatotic grafts were associated with higher 30-day mortality following transplantation [51].

However, the majority of them are experimental, and data is limited. Interestingly, a study showed that intravenous N-Acetyl Cysteine exposure 20 min prior and 1 h after reperfusion showed better ATP reappearance, bile secretion, and glutathione metabolism. Ex situ machine perfusion is being used as a preservation technique to decrease preservation injury and in addition to improve graft quality and survival, it also allows for assessment and treatment while preserving the organ [52]. Thus, Ex situ machine perfusion can contribute to defatting techniques, however direct treatment for steatosis has not been established yet [53]. Therefore, in conclusion, relevant factors such as age, degree of macro steatosis, and ischemia time should be considered when using marginal donors for better outcomes.

## 4. Elderly Donors

As per the United Network for Organ Sharing (UNOS) database, a continuous rise in old-age donors is reported. Donors with age > 65 years increased from 398 in 2000 to 675 in 2020 [54]. This likely resulted from the organ shortage due to the increasing number of patients requiring LT. Older grafts were considered suboptimal because at the cellular level with an increase in age, there is a decrease in the number of smooth endoplasmic reticulum, mitochondria, glutathione reductase (antioxidant) and cytochrome P450 (declined by 16% after 40 years and 32% after 70 years) [55,56]. However, it has been shown that because of its adequate reserve function, dual blood supply, and regenerative capacities, the synthetic function of the liver is not affected by age [57]. A retrospective study comparing donors of age ≥ 65 years (*n* = 50) with donors of age <65 years (*n* = 50) showed that there were no differences in histological changes in post-reperfusion biopsies, rate of acute rejection episodes, patient survival, and graft survivals after one year of follow up [58]. Similarly, Rodriguez Gonzales et al. reported favorable outcomes using 100 liver grafts of elderly donors with age > 60 years once factors such as cold ischemia time of <6 h and macro vesicular steatosis < 30% were taken into consideration [59]. Comparatively, some studies have shown lower graft survival with older donors if the grafts presented had steatosis or prolonged cold ischemia time (CIT) [60,61]. Cold storage for the elderly donor liver grafts has shown unfavorable histological results, whereas hepatocytes under Normothermic Machine Perfusion (closer to the physiological environment) showed decreased Ischemic/Reperfusion Injury although clinically the benefits yet to be determined [62].

Furthermore, elderly donors should be carefully selected if the recipient is HCV viremic and has not been treated with DAA’s. Historically, before DAAs were approved, Selzer et al. reported that donors above 45 years of age carry an eight times higher risk of progressing into advanced fibrosis by 2 years post-transplant [63]. This increasing trend of advanced fibrosis was seen to be linked with the progression of recurrent HCV. Similarly, a study involving 553 recipients, factors including donor age of 60 to 79 years, showed HCV positive recipients and MELD > 25 were related with poor survival [64]. HCV recurrence and ischemic type biliary injuries are significant factors leading to elderly donor transplantation failures, and since in current times, DAAs keep HCV under check, prevention of ischemia/reperfusion injuries can majorly determine the success of using elderly donor grafts [65,66,67]. In general, increased age also increases biliary complications and hepatic artery thrombosis [68,69].

Given the current evidence, utilizing grafts from elderly donors for increasing the donor pool is relatively safe. However, the donor selection should be carefully done, including donors with normal liver function, absence of atherosclerosis in the hepatic artery, satisfactory hemodynamics at pre harvesting state, lesser number of days in ICU, less CIT, macro steatosis < 30%, and no histological changes on biopsy. Also, when HCC is an indication for transplant, the risk for increased HCC recurrence with older grafts has to be considered [70]. This was demonstrated in a study showing higher rate of post-LT HCC recurrence when using donors above 60 years of age (adjusted hazard ratio = 1.38, 95% confidence interval 1.10 to 1.73, *p* = 0.006) [48].

## 5. Split Liver Grafts

Split liver transplantation (SLT) pioneered by Rudolf Pichlmayr in 1988 involves transplantation of liver from a single donor into two recipients, an adult and a pediatric patient [71]. In SLT, the liver is usually divided into a more extensive right tri-segment (segment I, IV to VIII) kept for adults and a smaller left lateral segment (segment II and III) reserved for children [72]. Split grafts are acquired from superior quality donors who show minimal other marginal factors. The creation of an anatomic variant post splitting and the time consumed in splitting as well as transportation prolongs CIT leading to less favorable results [73]. The prognosis is significantly affected when a particular type of splitting procedure is used. External liver graft splitting, because of being more time consuming (owing to longer transportation times) and higher surgical complications, was associated with worse outcomes than in-house liver graft splitting method [73]. The latter involves implantation of both left and right lobes at the same center where the splitting procedure is done, and this not only reduces transportation time but also ensures that the surgeon involved in splitting (better anatomic acquaintance) is involved in implantation as well [73]. Although earlier studies with SLT resulted in higher morbidity and mortality, progressively with newer technologies, better outcomes are being reported [74,75]. A comparative study showed no difference in 1 year graft/patient survival between split and whole graft recipients in adult (89% in both, 89% vs. 92%, respectively) and in pediatric recipients (90% vs. 97%, 94% vs. 97%, respectively) [76]. Similarly, a meta-analysis comparing whole LT (WLT) to SLT consisting of 17 studies involving 48,457 patients showed comparable patient and graft survival [77]. Even though SLT is establishing its place, it is associated with complications like higher rates of overall biliary complications, bile leaks, vascular complications, hepatic artery thromboses, and outflow tract obstructions [77]. In fact, subgroup analyses of the above-mentioned meta-analysis revealed that using ex vivo SLT was associated with increased biliary and vascular complications [77]. This is likely due to prolonged ischemia times with the ex vivo method. Whereas with in vivo it is a more controlled environment, and the use of intraoperative ultrasound and vascular clamping provides a better understanding of vascular and biliary drainage [78]. Thus, using the liver’s special ability to regenerate, SLT can be made more successful by carefully selecting the recipient, decrease CIT and adapting standardized splitting procedures. Reducing transportation times with better planning and coordination between hospital centers is another significant modifiable factor that can improve outcomes. It will help to increase the organ pool by bridging the liver demand-supply gap and decreasing waitlist mortality in the pediatric and adult population.

## 6. Donor Post Cardiac Death (DCD)

Transplantation from a donor post-cardiac death (DCD) is an attractive option for ameliorating organ shortages and decrease transplant waitlist mortality. Based on Transplant Data in the annual report of the US Organ Procurement and Transplantation Network, 5% of the liver graft pool in the US is from donors’ post-cardiac death. In contrast to the donation post brain death (DBD) organs, DCD livers suffer a loss of blood supply resulting in hypoxemia [79]. This warm ischemic time (WIT), which is the time between cardiac death and organ cooling while procuring them, is variable and likely determines the complication after LT of DCD organs [80,81]. Therefore, controlled donors, for example, patients awaiting cardiac arrest, are preferred for LT as WIT can be closely monitored. Additionally, methods such as hypothermic machine perfusion, widely used in kidney grafts, have been proposed for DCD. Guarrera et al. did a matched cohort study where hypothermic machine perfusion was used in 31 livers matched with a control group (static cold storage) [82]. The results showed comparable outcomes for graft dysfunction rates and 1-year patient survival. Furthermore, there were lesser biliary complications and shorter hospital stay in hypothermic machine perfusion group [82]. Regarding the effect of the preservative solution used during procurement, one retrospective study suggested biliary complications to be higher with Histidine-tryptophan-ketoglutarate solution when compared to University of Wisconsin solution and the study concluded that avoiding Histidine-tryptophan-ketoglutarate while using grafts from DCD could give better results [83].

A meta-analysis comprising of 25 studies with 62,184 LTs compared DCD with DBD. Results showed that with DCD grafts, there was a considerable rise in biliary complications (OR = 2.4 (1.9, 3.1); *p* < 0.00001), with significant decrease in 1 year (OR = 0.7 (0.5, 0.8); *p* = 0.0002) and 3 years (OR = 0.6 (0.5, 0.8); *p* = 0.001) graft survival [84]. A considerable fall in 3-year patient survival (OR = 0.7 (0.5, 1.0); *p* = 0.04) was also seen [84]. However, a recent metanalysis showed that there was considerable drop in ischemic type biliary lesions (ITBLs) (*p* = 0.04), re-transplantation rate (*p* = 0.0001), with longer 1 year graft survival (*p* = 0.02) among patients who received thrombolytic tissue plasminogen activator (tPA) flush in DCD LT [85]. From the data, it is evident that DCD LT results in high graft failure and biliary complication rates. However, due to the critical organ shortage, these grafts should not all be discarded. Another complication associated with DCD grafts is Acute Kidney Injury (AKI) and chronic kidney disease (CKD) as a sequela [86]. A higher incidence of AKI was reported with DCD grafts, and this was thought to be a consequence of ischemic reperfusion injury. Simultaneously elevated AST levels perioperatively supported this causation [86]. To summarize, the focus should be directed towards reducing WIT, CIT, and careful recipient selection, possibly amongst lower MELD patients. Appropriate postoperative care is paramount to improve overall results.

## 7. Donors with Benign Tumors of Liver

Benign tumors of the liver include cavernous hemangioma, perivascular epithelioid cell tumor, inflammatory pseudotumor, and focal nodular hyperplasia. The accuracy of MRI has made diagnosis of hepatic adenoma, focal nodular hyperplasia and hemangioma easy, thus limiting the use of biopsy [87]. For inflammatory pseudotumor, angiomyolipoma or abnormal underlying hepatic parenchyma, where imaging can be doubtful, routine use of liver biopsy is justified [87]. To increase the donor pool in times of emergent need, successful use of grafts from benign tumors has been reported. Li et al. reported 15 successful transplants with postoperative pathological diagnosis reported cavernous hemangioma (*n* = 11), perivascular epithelioid cell tumor (*n* = 2), inflammatory pseudotumor for (*n* = 1), and focal nodular hyperplasia (*n* = 1)) from grafts, which otherwise would have been discarded [52]. Similarly, grafts with inflammatory pseudotumor which appears to be radiographically similar to hepatocellular carcinoma (HCC) was successfully transplanted [88]. Likewise, cases of successful LT donors with cavernous hemangioma have been reported in literature [89,90]. A case series reported multiple successful LTs with otherwise discarded grafts with benign tumors and suggested the use of prosthetic vascular grafts for their hepatic venous flow reconstruction [52]. Therefore, data in support of using benign tumor grafts are emerging and what remains unknown is studies with a longer follow up time which could provide more clarity on safety in using such grafts.

## 8. Others

The accuracy of MRI donors are donor with ICU stay with ventilation >7 days, donor with serum sodium >165 mmol/L, transaminases: ALT > 105 U/L, AST > 90 U/L and serum bilirubin >3 mg/dL [91]. Furthermore, cold ischemia time within 8–10 h is considered favorable for liver graft [92]

## 9. Conclusions

Currently, LT is still restricted because of the shortage of available donor organs. Therefore, the novel concept of using marginal donors appears to be an option to partly meet the increasing organ demand. Although limited, currently available literature supports this notion. Therefore, in carefully selected individuals, with possible lower MELD scores, waitlist mortality may be avoided with the usage of marginal grafts. However, additional prospective studies with a larger sample size and longer follow-ups are required for standardization. Both donor and recipient characteristics have to be kept in mind while selecting the right organ for the right patient.
**Summary for High-Risk Donors in Liver Transplantation**❖Virally infected donor grafts:
1.HIV:HIV positive donors to HIV positive recipients with HAART under HIV Organ Policy Equity (HOPE) Act.2.HCV:HCV positive donors to HCV negative recipients-prompt pan-genotypic DAA therapy and minimizing other graft limiting factors like, allograft fibrosis less than stage 2 and young donor age.3.HBV:-HbsAg positive donors to HBV positive recipients-HBIG and anti-viral drugs-HbcAb positive donors to HBV positive recipients-only anti-viral drugs.❖Donors with Fatty Liver:
Micro steatosis of any degree can be safely used.Macro steatosis of mild degree (<30%) preferred.❖Elderly donors (>65 years):
Minimize other graft limiting factors to include normal liver panel, absence of atherosclerosis in the hepatic artery, satisfactory hemodynamics at pre harvesting state, lesser number of days in ICU, less CIT, macro steatosis <30%, and no histological changes on biopsy.❖Split liver grafts:
Improved outcomes with decreased CIT, adapting standardized splitting procedures, reducing transportation time with better planning and coordination between hospital centers.❖Donor post cardiac death:
Improved outcomes with reducing WIT, CIT, and careful recipient selection, possibly amongst lower MELD patients.❖Donor with benign tumour of liver:
Supporting data emerging, longer follow up studies awaited.

## Data Availability

No additional data available.

## Abbreviations

CIT	Cold Ischemia Time
DCD	Donor post Cardiac Death
DBD	Donor post Brain Death
DDLT	Deceased donor liver transplantation
DAA	Direct Acting Anti virals
ESLD	End stage liver disease
HBIG	Hepatitis B Immunoglobulin
HCC	Hepatocellular carcinoma
HOPE	HIV Organ Policy Equity
HAART	Highly Active Anti-Retroviral Treatment
LT	Liver Transplantation
LDLT	Live donor liver transplantation
MELD	Model for End-Stage Liver Disease
NAT	Nucleic Acid Testing
SLT	Split Liver Transplantation
SRTR	Scientific Registry of Transplant Recipients
WLT	Whole Liver Transplant
US	United States
UNOS	United Network for Organ Sharing
